# ^18^F-FDG PET can effectively rule out conversion to dementia and the presence of CSF biomarker of neurodegeneration: a real-world data analysis

**DOI:** 10.1186/s13195-024-01535-3

**Published:** 2024-08-13

**Authors:** Sébastien Heyer, Maïa Simon, Matthieu Doyen, Ali Mortada, Véronique Roch, Elodie Jeanbert, Nathalie Thilly, Catherine Malaplate, Anna Kearney-Schwartz, Thérèse Jonveaux, Aurélie Bannay, Antoine Verger

**Affiliations:** 1 Department of Nuclear Medicine and Nancyclotep Imaging Platform, Université de Lorraine, CHRU Nancy, Nancy, F-54000 France; 2 Department of Methodology, Promotion and Investigation, Université de Lorraine, CHRU-Nancy, Nancy, F-54000 France; 3 Université de Lorraine, IADI, INSERM U1254, Nancy, F-54000 France; 4 Department of Biochemistry, Université de Lorraine, CHRU-Nancy, Nancy, F-54000 France; 5 Department of Geriatrics, Université de Lorraine, CHRU-Nancy, Nancy, F-54000 France; 6 CMRR, University Hospital Nancy, Nancy, F-54000 France; 7 Department of Neurology, University Hospital Nancy, Nancy, F-54000 France; 8 Medical Assessment and Information Department, Université de Lorraine, CHRU-Nancy, Nancy, 54000 France

**Keywords:** Real-world data; national health data system, FDG PET, Neurodegenerative disease, Prognosis

## Abstract

**Background:**

Precisely defining the delay in onset of dementia is a particular challenge for early diagnosis. Brain [^18^F] fluoro-2-deoxy-2-D-glucose (^18^F-FDG) Positron Emission Tomography (PET) is a particularly interesting tool for the early diagnosis of neurodegenerative diseases, through the measurement of the cerebral glucose metabolic rate. There is currently a lack of longitudinal studies under real-life conditions, with sufficient patients, to accurately evaluate the predictive values of brain ^18^F-FDG PET scans. Here, we aimed to estimate the value of brain ^18^F-FDG PET for predicting the risk of dementia conversion and the risk of occurrence of a neurodegenerative pathology.

**Methods:**

Longitudinal data for a cohort of patients with no diagnosis of dementia at the time of recruitment referred by a tertiary memory clinic for brain ^18^F-FDG PET were matched with (1) data from the French National Health Data System (NHDS), (2) data from the National Alzheimer Bank (NAB), and (3) lumbar puncture (LP) biomarker data. The criteria for dementia conversion were the designation, within the three years after the brain ^18^F-FDG PET scan, of a long-term condition for dementia in the NHDS and a dementia stage of cognitive impairment in the NAB. The criterion for the identification of a neurodegenerative disease in the medical records was the determination of LP biomarker levels.

**Results:**

Among the 403 patients (69.9 ± 11.4 years old, 177 women) from the initial cohort with data matched with the NHDS data, 137 were matched with the NAB data, and 61 were matched with LP biomarker data. Within three years of the scan, a ^18^F-FDG PET had negative predictive values of 85% for dementia conversion (according to the NHDS and NAB datasets) and 95% for the presence of LP neurodegeneration biomarkers.

**Conclusion:**

A normal brain ^18^F-FDG PET scan can help rule out the risk of dementia conversion and the presence of cerebrospinal fluid (CSF) biomarker of neurodegeneration early with high certainty, allowing modifications to patient management regimens in the short term.

**Trial registration:**

Clinical Trials database (NCT04804722). March 18, 2021. Retrospectively registered.

**Supplementary Information:**

The online version contains supplementary material available at 10.1186/s13195-024-01535-3.

## Background

Neurodegenerative pathologies, particularly Alzheimer’s disease (AD), are becoming increasingly common due to both the general aging of the population and improvements in the management of other comorbidities that increase the longevity of at-risk individuals. Neurodegenerative diseases are a major public health problem, resulting in a loss of autonomy for many patients as well as generating significant costs (estimated at 818 billion dollars in 2015) for diagnosis and management of patients [1]. Pathophysiological models of neurodegenerative diseases, particularly those of AD, describe these diseases as progressive conditions with asymptomatic or mildly symptomatic phases of varying durations (for example, subjective cognitive impairment (SCI) and mild cognitive impairment (MCI)) before conversion to dementia [2].

Precisely defining the delay in onset of dementia is a particular challenge for early diagnosis. The yearly risk of objective cognitive impairment in patients without cognitive complaints is estimated to be 8%, and the yearly risk of conversion to dementia among patients with MCI is estimated to be 22% [3]. Identifying patients who will convert to dementia is essential to be able to develop specific management regimens early [4, 5], to develop future therapeutics [6], and to limit the repetition of unnecessary, often invasive and/or expensive complementary examinations in patients at low risk of conversion.

The etiological diagnosis of neurodegenerative disease relies on clinical criteria and involves complementary examinations such as magnetic resonance imaging (MRI) for making a differential diagnosis and cerebrospinal fluid (CSF) biomarker assays or positron emission tomography (PET) scans for making a positive diagnosis. Currently, postmortem autopsy remains the only way to confirm a diagnosis of a neurodegenerative disease [2, 7].

Notably, brain [^18^F] fluoro-2-deoxy-2-D-glucose (^18^F-FDG) PET is a particularly interesting tool for the early diagnosis of neurodegenerative diseases [8], especially AD, as it can provide information about neuronal activity through the measurement of the cerebral glucose metabolic rate (CGMr). Brain ^18^F-FDG-PET is recommended to support early diagnosis of AD in MCI, but also early diagnosis of dementia with Lewy bodies and frontotemporal lobar degeneration [9]. However, the importance of brain ^18^F-FDG PET in the diagnosis of neurodegenerative diseases, especially AD, is currently debated in light of the diagnostic performance of CSF biomarkers, the advent of amyloid and tau PET radiotracers and the recent development of plasma biomarkers [10, 11]. This imaging modality has nevertheless been shown to predict the progression of MCI to AD dementia [12] as well as cognitive decline in patients without dementia [13]. The studies aiming to predict conversion to dementia with ^18^F-FDG PET are nonetheless scarce, not always combining visual and semi-quantitative analyses as recommended in clinical routine [14], and with outcomes mainly based on diagnostic follow-ups only [12]. Therefore, there is currently a lack of longitudinal studies under real-life conditions, with sufficient patients, to accurately evaluate the predictive values of brain ^18^F-FDG PET scans for the risk of conversion to dementia [12, 15, 16].

The main objective of this study was to use longitudinal, real-world data to estimate the prognostic value of brain ^18^F-FDG PET scans for the risk of conversion to dementia in SCI and MCI patients and the associated factors within the three years following the PET scan. Additionally, the secondary objectives included the prognostic value of brain ^18^F-FDG PET scans in determining the risk of occurrence of a neurodegenerative pathology, particularly AD, in determining the risk of death, and in identifying factors associated with the brain ^18^F-FDG PET scan results. For these purposes, the brain ^18^F-FDG PET scan results were matched with data from the French National Health Data System (NHDS), the National Alzheimer’s Bank (NAB) and medical records. There is a real need to link PET imaging data with real-world databases in large cohorts of patients to better demonstrate the predictive values of brain ^18^F-FDG PET imaging.

## Methods

### Participants

In this study, data were used from a longitudinal cohort of consecutive patients referred by a tertiary memory clinic (Centre Mémoire de Ressources et de Recherche (CMRR)) for a brain ^18^F-FDG PET scan at the Centre Hospitalier Régional Universitaire (CHRU; Regional University Hospital) of Nancy to determine whether evidence of a neurodegenerative pathology was present. Patients who obtained scans between January 1, 2010, and January 1, 2019, were retrospectively included in this study (ClinicalTrials.gov number NCT04804722). The prescription of a brain ^18^F-FDG PET scan by the tertiary memory clinic followed the national French recommendations for the diagnosis of AD and related disorders in force at this time period [10].

All included patients presented with cognitive complaints. A consultation with > 5 years of experience neurologist/geriatrician and neuropsychological assessment at the tertiary memory clinic, as well as brain morphological imaging (MRI or tomodensitometry), were performed before the brain ^18^F-FDG PET scan. Patients were followed longitudinally for at least three years until January 1, 2022.

This study was granted approval by the National Comité Ethique et Scientifique pour les Recherches, les Etudes et les Evaluation en Santé (CESREES, file no. 4,611,320 Bis) and by the Commission Nationale Informatique et Libertés (CNIL, Decision number: DR-2022-090) and was conducted following the principles of the Declaration of Helsinki. This trial was also reported in the Clinical Trials database (NCT04804722). The STROBE statement [17] was used as a reporting guide for the present article.

### PET acquisition and reconstruction

Prior to 2018, brain ^18^F-FDG PET images were acquired on an analog system (Biograph 6, Siemens^®^); thereafter, a digital system (Vereos, Philips^®^) was used.

Following an injection of 4.5 MBq/kg (for the analog device) or 2 MBq/kg (for the digital device) of ^18^F-FDG, the patient underwent neurosensory rest for 30 min. Patients were scanned in a supine position with a single bed position for an acquisition time of 10 min with the analog system and 15 min with the digital system. All patients were instructed to fast for at least six hours before the injection and had a blood glucose level < 10 mmol/L.

Image reconstruction was performed using the Ordered Subset Expectation Maximization (OSEM) iterative reconstruction algorithm. The reconstruction was conducted over two iterations with 21 subsets, a 256 × 256 matrix, 2.7 × 2.7 × 2.7 mm^3^ voxel spacing and postfiltering with a 4-mm Gaussian filter for the analog PET device [18] and over two iterations with 10 subsets, a 256 × 256 matrix, 1 × 1 × 1 mm^3^ voxel spacing and point spread function (PSF) correction for the digital PET device [19]. Corrections for attenuation, random coincidences and dispersion were applied for the images obtained with both devices.

### PET image analysis

**Fig. 1:**
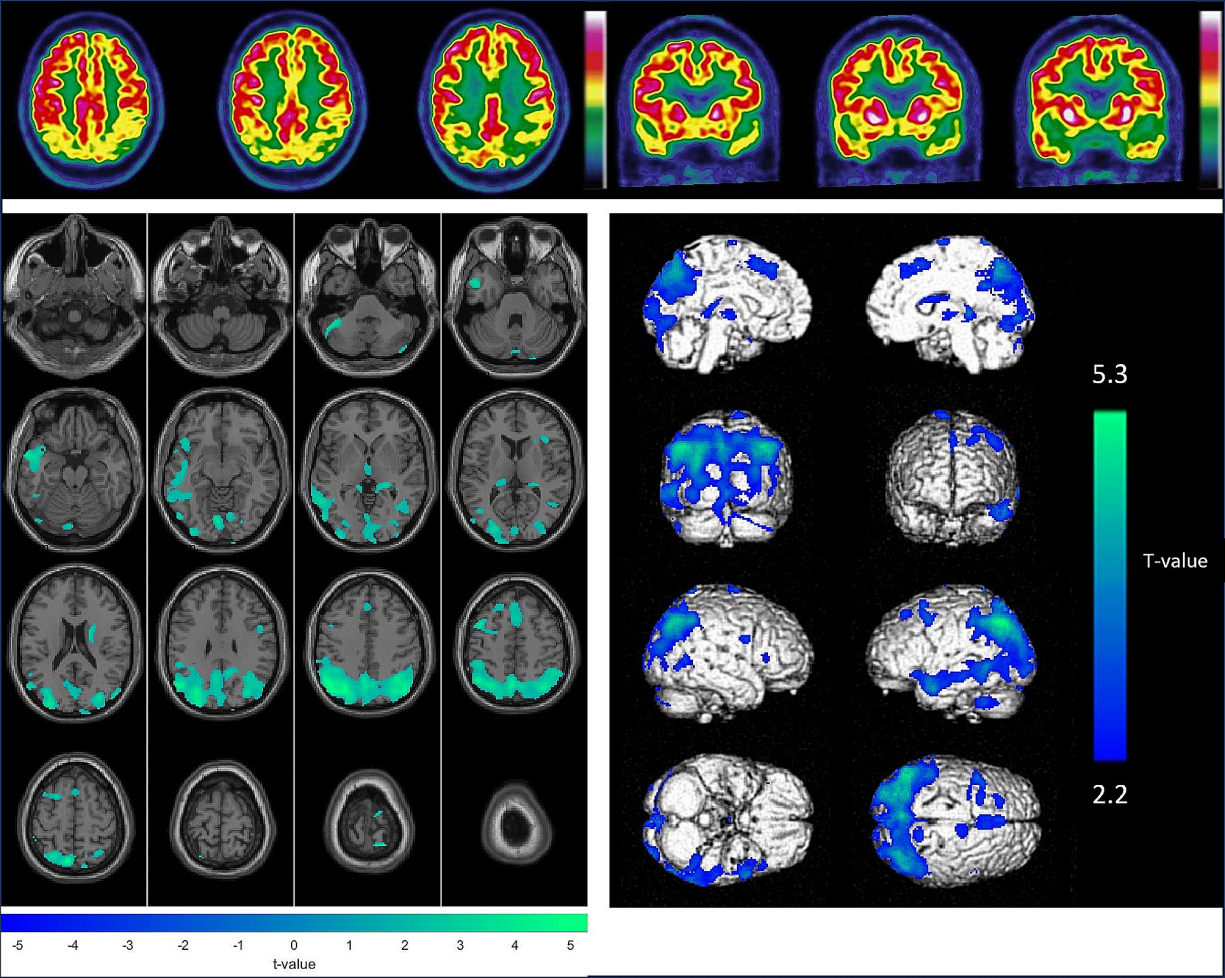
Example of the combined visual (upper panel, axial and coronal slices of brain ^18^F-FDG PET images) and semiquantitative (lower panel, with hypometabolisms in green projected on axial MR slices on the left and hypometabolisms in blue on a 3D volume rendered on the right) analysis for a 65-year-old male patient (initially diagnosed with MCI); these results were consistent with Alzheimer’s disease. The final diagnosis in the National Alzheimer’s database was Alzheimer’s disease and conversion to dementia after 31.5 months

A combined visual and semiquantitative analysis of the brain ^18^F-FDG PET images was performed by two experienced physicians (S.H. and A.V.) who were blinded to all clinical data, with final consensus in case of discordances. This combined analysis was performed according to the latest guidelines of the European Association of Nuclear Medicine [9]. First, the brain ^18^F-FDG PET scans were reviewed visually based on the typical regions of hypometabolism associated with neurodegenerative pathologies accurately defined in the latest European Association of Nuclear Medicine guidelines [9]. The semiquantitative analysis of the brain ^18^F-FDG PET images was performed by an experienced engineer (M.D.) using the Statistical Parametric Mapping software, SPM 12, run on MATLAB 2020b (MathWorks, Inc., Sherborn, MA). Brain ^18^F-FDG PET images were spatially normalized using a dementia-specific ^18^F-FDG-PET template [20] and intensity-normalized with proportional scaling. All images were subsequently smoothed with an 8 mm full-width at half-maximum (FWHM) Gaussian filter. Statistical analyses of this semi-quantitative analysis were performed individually at the voxel-to-voxel level according to an optimized procedure validated by Perani et al. [21] via comparison to a dataset of healthy participants from the Alzheimer’s Disease Neuroimaging Initiative (ADNI) cohort (*n* = 75; age (mean ± SD) 72.25 ± 5.25; 42 women). Age was treated as a continuous variable and used as a covariate. A Z score map of individual hypometabolism was calculated for each scan via the two-sample Student’s t test. A probability threshold of *p* < 0.05 was considered to indicate statistical significance, and the extent threshold (k extent) was set at 100 voxels. An illustrative example of this process is available in Fig. 1.

In both the visual and semiquantitative analyses, the brain ^18^F-FDG PET scans were classified as normal, abnormal but incompatible with a neurodegenerative disease diagnosis, or abnormal and compatible with a neurodegenerative disease diagnosis. Brain ^18^F-FDG PET scans deemed compatible with a neurodegenerative pathology were further divided into two categories: those compatible with an AD pattern and those that were not according to the hypometabolic areas described in the pattern of AD in the latest European Nuclear Medicine Association guidelines [9].

### Real-world outcome data collection, sources, and linkage methods

The patient cohort was matched with three databases representing patient data obtained under real-life conditions: the NHDS, a database to which any general practitioner, whether practicing in or outside a hospital setting, can submit data and therefore includes the largest cohort among the three databases; the NAB, a more specific database to which only neurologists and geriatric specialists can submit data; and, finally, data from objective CSF AD biomarker tests. The French NHDS makes possible, since 2016, to link health insurance data, hospital data, and more recently the medical causes of death, disability-related data, and a sample of data from complementary health insurance organizations. The purpose of the NHDS is to make these data available in order to promote studies, research or evaluations of a nature in the public interest. The NAB is a French central information system of the memory clinic network that coordinates the management of patients with neurodegenerative disorders according to the 2009 national guidelines. This national database includes longitudinal follow-up data for patients who attended a tertiary memory clinic for cognitive complaints. These data include information obtained through initial and follow-up visits and syndromic and etiological diagnoses. Each participating center is required to provide information on patients seen for cognitive complaints through a computer file with limited space for data entry to facilitate and improve participation in the national database. The CSF AD biomarker tests were retrieved from medical records of the Nancy University Hospital. These CSF biomarkers allow to define AD biologically according to the A/T/N classification [22, 23].

The patient cohort was first matched with the patients in the NHDS to identify those who experienced a conversion to dementia within the three years after the brain ^18^F-FDG PET scan. As the NHDS does not collect identifiable patient information (i.e., surname, family name, health insurance identification number, date of birth), a deterministic indirect data linkage method was applied between data from the patient cohort and those from the anonymized NHDS based on common variables (sex, month and year of birth, identification number in the Regional University Hospital of Nancy, date of brain ^18^F-FDG PET scan, and date of lumbar puncture (LP), when available). This method could result in potential incorrect matches. However, incorrect matches were limited as we retained only patients with a 1:1 match, i.e., patients with multiple correspondence in one or the other database were excluded. Patients designated as having a “long-term condition” (LTC) of dementia at the time the brain ^18^F-FDG PET scan was performed were secondarily excluded.

Data for patients in the cohort who matched with those in the NHDS and presented without dementia at the time of brain ^18^F-FDG PET scanning were then matched with the clinicobiological data obtained in real-life conditions from the NAB and with the medical records of the Nancy University Hospital. These data could be directly linked by matching patient name, surname, sex, and date of birth.

### Outcomes, endpoints and extracted variables

The main outcome was conversion to dementia according to the data in the NHDS and NAB within three years of the brain ^18^F-FDG PET scan, verifying that this information was concordant between these two databases. The secondary outcomes were a diagnosis of a neurodegenerative disease according to the NAB data and medical records, death according to the NHDS data, and factors associated with a conversion to dementia and brain ^18^F-FDG PET results according to the NHDS and NAB data.

The criterion for dementia conversion in the NHDS was the designation of an LTC for dementia by any general practitioner within the three years after the brain ^18^F-FDG PET scan. Aside from age, sex and educational level, which were extracted from the brain ^18^F-FDG PET scan records when available and are known factors influencing cognitive decline, data collected from the NHDS including the designation of an LTC for dementia, and other factors influencing conversion to dementia such as hospitalization and death within the three years after the PET scan, and other LTC designations and medications potentially related to neurodegenerative pathologies.

The criterion for conversion to dementia in the NAB was the identification of a dementia stage of cognitive impairment within the three years after the brain ^18^F-FDG PET scan. The criterion for the diagnosis of a neurodegenerative disease was a final diagnosis at the last follow-up consultation in the NAB within the three years after the brain ^18^F-FDG PET scan. Data concerning the stage of cognitive impairment (initial and during follow-up) and syndromic (dysexecutive, amnesic, linguistic, and diffuse) and etiological diagnoses (grouped into five classes: neurodegenerative diseases, psychiatric pathology, vascular damage, other causes (i.e., epilepsy, encephalopathy/encephalitis, intracranial tumor, posttraumatic sequelae and other organic causes), and subjective memory complaints) at the last follow-up consultation were also collected from the NAB.

The criterion for the identification of a neurodegenerative disease in the medical records was the determination of LP biomarker levels according to the A/T/N classification [22, 23] according to laboratory reference values, i.e., A + if Aß42 < 700 pg/mL or Aß42/Aß40 < 0.06, T + if pTau > 60 pg/mL and N + if tTau > 350 pg/mL. All CSF biomarker assays were performed in the same laboratory at Nancy University Hospital using the manual Innotest ELISA technique with 10 mL polypropylene sampling tubes (Ref: TP 10 − 03 GOSSELIN (CML)).

### Statistical analyses

Categorical variables are expressed as numbers and percentages, and continuous variables are expressed as means and standard deviations or medians and quartiles. According to the NHDS, the sensitivity (Se), specificity (Sp), accuracy (Acc), positive predictive value (PPV), and negative predictive value (NPV) of brain ^18^F-FDG PET scans classified as supporting or not supporting the diagnosis of a neurodegenerative disease were calculated for the risk of dementia conversion within three years. Because all-cause deaths might interfere with the identification of dementia conversion, we identified three groups of patients: patients who did not progress to dementia and were still alive during the three years following the PET scan, patients who progressed to dementia before dying or were still alive during the follow-up period, and patients who died without having been documented as converting to dementia.

We conducted bivariate and multivariable analyses with stepwise selection to identify predictive factors for dementia conversion within the three years following the PET scan using the Fine and Gray model for competing risk, with all-cause death as the competing risk for dementia conversion. We also performed a subanalysis to identify predictive factors only for patients whose brain ^18^F-FDG PET scans did not support the diagnosis of a neurodegenerative disease using bivariate and multivariable logistic regressions. Variables with a *p* value < 0.1 in bivariate analyses were selected as candidates for the multivariable model. Spearman correlation was assessed between candidate variables with a threshold of *R* > 0.75. Variables occurring fewer than five times were not included in the multivariable analyses. The diagnostic performance metrics for brain ^18^F-FDG PET scans for the risk of death within three years were also calculated. Kaplan‒Meier analysis with log rank tests was conducted to determine the prognostic value of brain ^18^F-FDG PET for dementia-free survival and overall survival.

The Se, Sp, Acc, PPV and NPV of brain ^18^F-FDG PET scans classified as supporting or not supporting the diagnosis of a neurodegenerative disease were also calculated for the risk of dementia conversion within three years with respect to the NAB and the medical records from the Nancy University Hospital. In addition, a bivariate logistic regression analysis was performed to assess the value of the brain ^18^F-FDG PET scan interpretation as well as age, sex and educational level for the risk of conversion to dementia within three years according to the NAB data. These PET diagnostic performance metrics were also calculated for the diagnosis of any neurodegenerative pathology, for the specific diagnosis of AD (according to the NAB data), and for detecting different combinations of CSF biomarkers (according to the medical records of Nancy University Hospital). The NAB data and medical records were also subjected to subanalyses involving PET scans demonstrating a pattern that favored or did not favor an AD diagnosis and only in the MCI patient group. Chi-square tests for the association of brain ^18^F-FDG PET scan interpretations with the initial stage of impairment and syndromic and etiological diagnoses were performed according to the NAB data.

Unless otherwise indicated, a *p* value < 0.05 was considered to indicate statistical significance. Missing data were not included in the analyses. All the statistical tests were performed with IBM SPSS 25.0 software and SAS Enterprise Guide version 8.2 (SAS Institute, Inc., Cary, N.C., USA).

## Results

### Patient numbers, characteristics, and matching rates

**Fig. 2:**
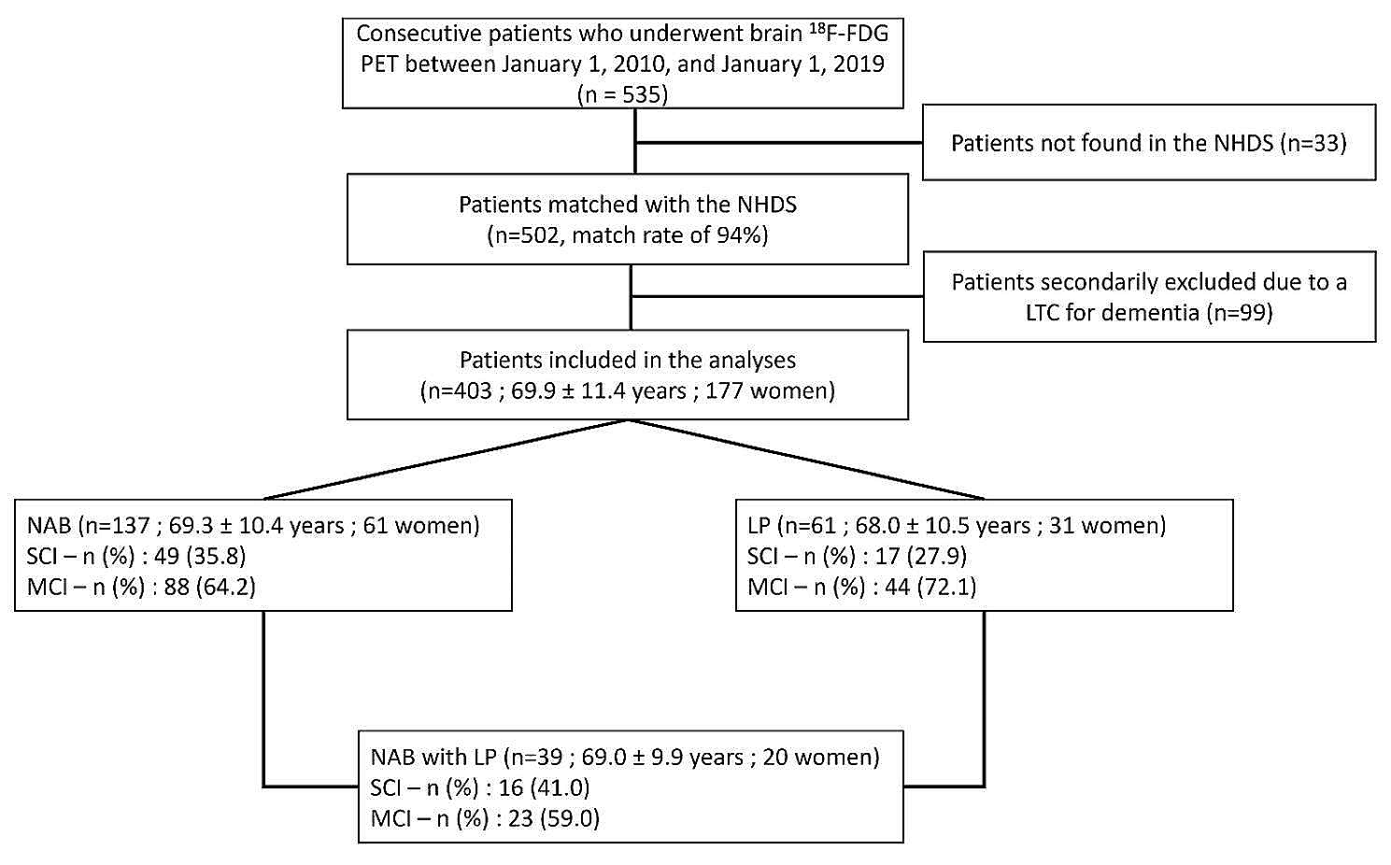
Flowchart for the inclusion of patients in this study. MCI, mild cognitive impairment; LP, lumbar puncture; LTC, long-term condition; NAB, National Alzheimer Bank; NHDS, National Health Data System; SCI, subjective cognitive impairment

Five hundred thirty-five patients were initially identified in the longitudinal patient cohort (Fig. 2). Among them, 502 were matched to the data in the NHDS (match rate 94%). Ninety-nine patients were secondarily excluded due to a recorded LTC for dementia at the time the brain ^18^F-FDG PET scan was performed. Four hundred and three patients (69.9 ± 11.4 years old, 177 women) ultimately fulfilled the inclusion criteria and were included in the analyses. Within the three years after the PET scan, 105 (26%) patients converted to dementia, 12 (3%) of whom died; 52 (13%) died without any dementia conversion; and 246 (61%) were still alive and did not experience any dementia conversion. The total median follow-up time according to Kaplan–Meier curves was 3.84 years [3.17; 4.61]. All data extracted from the NHDS are reported in Supplemental Table 1.

Among the 403 patients, matches with data in the NAB database were found for 137 (34%; 69.3 ± 10.4 years old; 61 women). Over a median follow-up of 1.39 years [0.69; 2.54], 26 (19%) patients in the NAB were classified as having SCI, 72 (53%) as having MCI, and 39 (29%) as having experienced a conversion to dementia (14 and 25 patients initially classified as having SCI and MCI, respectively). Moreover, at follow-up, 29 patients had a diagnosis of AD. At follow-up, the predominant syndromic diagnoses were amnesic in 42% (*n* = 58), dysexecutive in 20% (*n* = 28), linguistic in 14% (*n* = 19), and diffuse in 18% (*n* = 25) of the patients. The etiological diagnosis was neurodegenerative disease in 46% (*n* = 63), psychiatric disease in 22% (*n* = 30), vascular disease in 10% (*n* = 14), subjective memory complaints in 4% (*n* = 6) and other causes (epilepsy, encephalopathy/encephalitis, intracranial tumor, posttraumatic sequelae, and other organic causes) in 13% (*n* = 18) of the patients.

Among the 403 patients, 61 (15%) (68.0 ± 10.5 years old, 31 women) had undergone a LP for AD biomarker assays (39 of whom were included in the NAB). Of the 61 patients, 21 (34%) were classified as having the A+/T + profile (among whom only one also had the N + profile), and 7 (12%) had the N + profile alone. The median time between the PET scan and LP was 2 days [-124; 58].

A flowchart of the included patients is available in Fig. 2.

### Combined brain ^18^F-FDG PET analysis

After assessing the patient profiles in terms of dementia status, the presence of a neurodegenerative diagnosis, and LP profile, we sought to analyze the PET imaging findings of the patients in the cohort to determine their value in predicting the above characteristics. Final consensus of both visual and semi-quantitative analyses was needed in less than 10% of cases. Combined visual and semiquantitative analyses were performed on the 403 patients, revealing that 120 (30%) brain ^18^F-FDG PET scans were normal, two (< 1%) were abnormal but incompatible with a neurodegenerative disease, and 281 (70%) were classified as neurodegenerative, including 107 (38%) with a pattern consistent with AD.

Among the 137 patients whose data matched with those in the NAB, 52 (38%) had a normal brain ^18^F-FDG PET scan, and 84 (61%) had a scan classified as having a neurodegenerative pattern; among these, 25 (30%) showed a pattern compatible with AD. Among the patients who had undergone a LP, 18 (30%) had a normal brain ^18^F-FDG PET scan, and 42 (69%) had scans classified as neurodegenerative, including 12 (29%) with scans compatible with AD.

The number of normal brain ^18^F-FDG PET scans, which represents about 30% of cases, in patients with cognitive complaints who are addressed by a tertiary memory center, is thus not infrequent regardless the database queried.

### Performance metrics of brain ^18^F-FDG PET

In this first paragraph, we report the results to respond to our primary objective. Based on the outcomes reported by the NHDS, the brain ^18^F-FDG PET scans achieved a Se of 83%, Sp of 35%, Acc of 47%, PPV of 31% and NPV of 85% in predicting a conversion to dementia within three years of the PET scan. Among the 137 patients matched with the NAB data, the brain ^18^F-FDG PET scans had Se of 79%, Sp of 46%, Acc of 55%, PPV of 37% and NPV of 85% in predicting a conversion to dementia (Table 1). When restricting this analysis to MCI patients (*n* = 88), brain ^18^F-FDG PET scans metrics were Se of 80%, Sp of 43%, Acc of 53%, PPV of 36% and NPV of 84%.

**Table 1:** Brain ^18^F-FDG PET diagnostic performance

		PET scans showing a neurodegenerative pattern	PET scans showing an Alzheimer’s disease pattern
**Risk of conversion to dementia**	Se	79%	28%
	Sp	46%	86%
	PPV	37%	44%
	NPV	85%	75%
	Acc	55%	69%
**Diagnosis of neurodegenerative disease**	Se	84%	33%
	Sp	58%	95%
	PPV	63%	84%
	NPV	81%	63%
	Acc	70%	66%
**Diagnosis of Alzheimer’s disease**	Se	83%	62%
	Sp	44%	94%
	PPV	29%	72%
	NPV	91%	90%
	Acc	53%	87%

Diagnostic performance of brain ^18^F-FDG PET in predicting the risk of conversion to dementia and in diagnosing neurodegenerative disease and Alzheimer’s disease (with respect to data from the National Alzheimer Bank, *N* = 137). Abbreviations: Se, sensitivity; Sp, specificity; PPV, positive predictive value; NPV, negative predictive value; Acc, accuracy

**Fig. 3:**
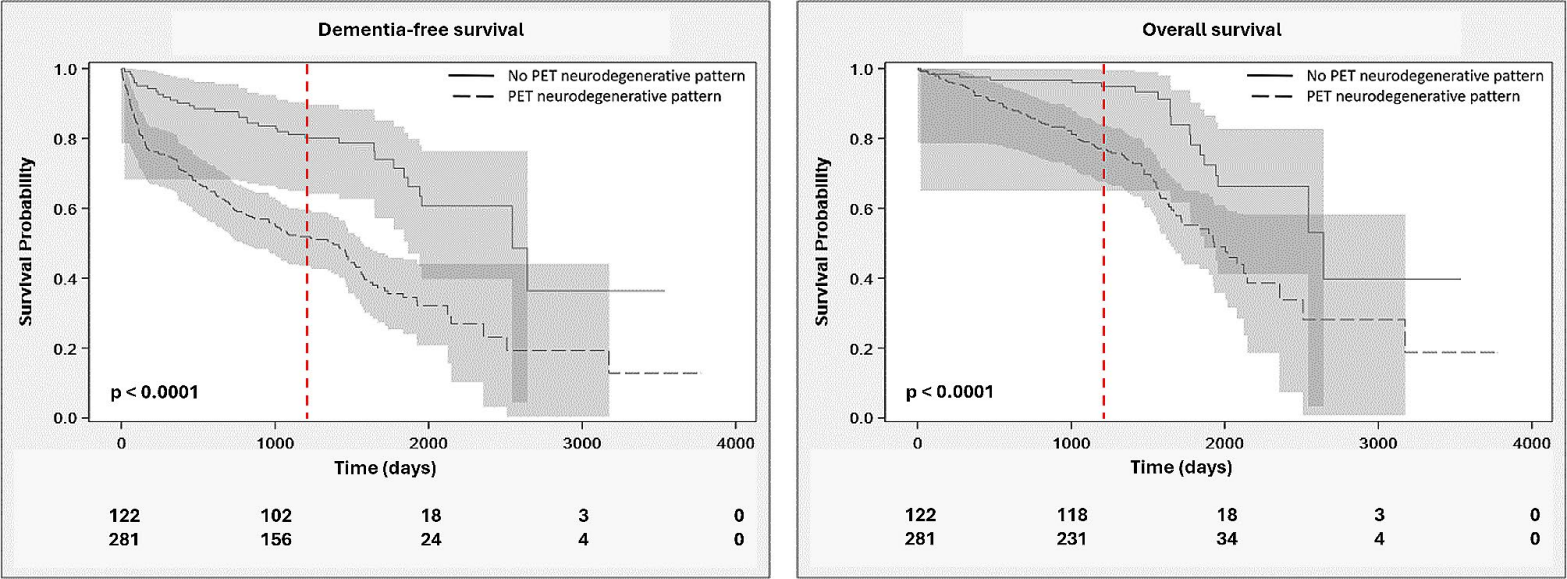
Dementia-free (left panel) and overall (right panel) survival curves and their 95% confidence intervals for patients with PET images demonstrating patterns supporting and not supporting a diagnosis of a neurodegenerative disease (survival according to the National Health Data System, *N* = 403). The red dotted lines indicate the 3 years threshold of follow-up to respond to the primary and one secondary objectives. Numbers below the respective figures represent the number of patients under follow-up without any dementia (left panel) or alive (right panel) with a PET scan not in favor of a neurodegenerative pattern (first row) and in favor of a neurodegenerative pattern (second row)

One of our secondary aims was to further assess the value of brain PET imaging in predicting other conditions related to a conversion to dementia, including mortality and survival (both dementia-free and overall). With respect to the NHDS data, the brain ^18^F-FDG PET scans demonstrated a Se of 92%, Sp of 35%, Acc of 44%, PPV of 21% and NPV of 96% in predicting death within the three years following the PET scan. Dementia-free survival significantly differed between patients whose brain ^18^F-FDG PET scans did or did not support a diagnosis of a neurodegenerative disease (45.4 [9.7; 77.4] months vs. 83.6 [51.2; not reached] months, *p* < 0.001; Fig. 3); a similar finding was observed for overall survival (63.4 [44.1; 104.2] months vs. 86.8 [61.3; not reached] months, *p* < 0.001; Fig. 3).

Another secondary objective was to determine the brain ^18^F-FDG PET scan metrics to identify neurodegenerative diseases. Using the NAB data as a reference, the brain ^18^F-FDG PET scans had an NPV of 81% in diagnosing neurodegenerative disease (91% for AD); when the analyses were restricted to MCI patients, the NPV was 78% (88% for AD). Among the other metrics, the Acc of the combined visual and semiquantitative analyses of the brain ^18^F-FDG PET scans was 70% in the diagnosis of neurodegenerative pathologies and 87% in the diagnosis of AD. All the diagnostic performance metrics of the brain ^18^F-FDG PET scans are summarized in Table 1. Regarding the Nancy University Hospital medical records, the brain ^18^F-FDG PET scans of the patients who underwent LP had an NPV of 95% in predicting the N biomarker profile and 79% in predicting the combination A and T biomarker profile. Restricting these analyses to MCI patients (*n* = 44), brain ^18^F-FDG PET had an NPV of 100% in identifying the N biomarker profile and 71% in identifying the combined A and T biomarker profile. The detailed results of the brain ^18^F-FDG PET scan performance metrics are detailed in Table 2.

It is important to note regarding these results that brain ^18^F-FDG PET scans present high NPVs for determining the risk of conversion to dementia as well as for the risk of death and the diagnosis of a neurodegenerative disease.

**Table 2:** Diagnostic performance of brain ^18^F-FDG PET in predicting CSF biomarker patterns

	PET scans showing a neurodegenerative pattern	PET scans showing an Alzheimer’s disease pattern
**A**+		
Se	76%	31%
Sp	38%	91%
PPV	52%	75%
NPV	63%	69%
Acc	56%	62%
**T+**		
Se	74%	26%
Sp	44%	94%
PPV	76%	92%
NPV	42%	35%
Acc	66%	46%
**N+**		
Se	86%	29%
Sp	33%	81%
PPV	14%	17%
NPV	95%	90%
Acc	39%	75%
**A+/T+**		
Se	81%	38%
Sp	38%	90%
PPV	40%	67%
NPV	79%	73%
Acc	52%	72%

Data from patient medical records (*N* = 61). Abbreviations: Se, sensitivity; Sp, specificity; PPV, positive predictive value; NPV, negative predictive value; Acc, accuracy; A for amyloid biomarker, T for tau biomarker and N for neurodegeneration biomarker

### Predictive and associative factor analyses

In addition to the brain ^18^F-FDG PET scans, we sought to identify clinical variables that also had predictive value for the conversion to dementia and the diagnosis of neurodegenerative diseases. We subjected different variables from the NHDS and NAB datasets, including variables detailed in Supplemental Table 1 for the NHDS and age, sex and educational level for the NAB, to bi- and multivariate analyses to identify independent predictive factors for these conditions. According to the NHDS data, age was the sole predictive factor of dementia conversion within 3 years for both the entire cohort (bivariate analysis in Supplemental Table 2) and when the analysis was restricted to patients with a PET scan with a neurodegenerative pattern (bivariate analysis in Supplemental Table 3). Furthermore, brain ^18^F-FDG PET scans supporting a diagnosis of AD only showed a tendency toward being a predictive factor for dementia conversion (*p* = 0.07 in multivariate analysis). In contrast, age under 60 years and a higher education level were the two predictive factors associated with obtaining a brain ^18^F-FDG PET scan that did not support diagnosis of a neurodegenerative disease (the only multivariable analysis showing at least 2 significant covariables is in Table 3, bivariate analyses in Supplemental Table 4).

**Table 3:** Factors associated with a normal brain ^18^F-FDG PET scan

Variable	Conditions	Patients with normal brain FDG-PET (*N* = 120) *n* (%)	Odds ratio	Confidence interval	*p* value
**Age**	< 60 years	48 (40.00%)	7.375	[3.121; 17.423]	**< 0.0001**
	60–69 years	30 (25.00%)	1.486	[0.665; 3.320]	0.334
	70–79 years	26 (21.67%)	1.359	[0.630; 2.929]	0.434
	> 80 years	16 (13.33%)			**-**
**Level of education**	Primary school	15 (14.42%)			**-**
	College	10 (9.62%)	0.944	[0.342; 2.604]	0.912
	Youth training NVQ (National Vocational Qualification)	16 (15.38%)	0.750	[0.312; 1.804]	0.521
	High school	25 (24.04%)	2.154	[0.905; 5.128]	0.083
	Graduate studies	38 (36.54%)	2.130	[1.026; 4.421]	**0.043**
	Missing data	16	-	-	-

Normal brain ^18^F-FDG PET scan: those that do not support a diagnosis of a neurodegenerative disease. Factors associated with a normal brain FDG-PET according to the National Health Data System: multivariable logistic regression analysis (*N* = 403)

According to the NAB data, the visual and semiquantitative interpretation of the brain ^18^F-FDG PET scans was the sole prognostic factor for dementia conversion (*p* < 0.01 in the multivariable analysis with age, sex, and education as covariates, *p* = 0.05 when restricting the analysis to MCI patients). Significant associations (*p* < 0.001) were found between brain ^18^F-FDG PET scans consistent with neurodegenerative disease and disease stage during follow-up (54% in SCI patients, 54% in MCI patients, and 79% in patients with dementia), syndromic diagnoses (46% in patients with dysexecutive impairment, 57% in patients with an amnestic diagnosis, 89% in patients with a linguistic diagnosis, and 80% in patients with a diffuse diagnosis), and etiological diagnoses (84% in patients with neurodegenerative impairment, 43% in patients with psychiatric pathologies, 57% in patients with vascular impairment, 33% in patients with other etiological causes of the diagnosis (epilepsy, encephalopathy/encephalitis, intracranial tumor, posttraumatic sequelae and other organic causes), and 0% in patients with subjective memory complaints).

Concerning predictive and associated factors, age is an important clinical factor to consider when evaluating the risk to conversion to dementia in the NHDS. Interestingly, brain ^18^F-FDG PET in favor of a neurodegenerative disease is also an important finding related to this outcome, since it was the sole independent factor in the NAB database when associated to clinical variables to determine the risk of conversion to dementia. Furthermore, high associations between brain ^18^F-FDG PET results and disease stage, syndromic and etiological diagnoses were observed.

## Discussion

This study primarily aimed to estimate the prognostic value of brain ^18^F-FDG PET scan for the risk of conversion to dementia with real-world data in a large cohort of patients addressed by a tertiary memory center, to accurately define the brain ^18^F-FDG PET metrics in this setting. In the current study, we responded to our main objective with brain ^18^F-FDG PET scans achieving a high NPV in predicting a conversion to dementia in routine clinical practice (85%). Moreover, high NPVs of brain ^18^F-FDG PET in predicting the diagnosis of neurodegenerative disease, assessing through objective biomarkers, i.e. a CSF N biomarker profile and the combined A/T biomarker profile in the diagnosis of AD (95% and 79%, respectively) were also observed, responding to a secondary objective, and confirming the potential of brain ^18^F-FDG PET to accurately predict the risk of neurodegenerative disease. Based on these diagnostic performances, brain ^18^F-FDG PET, depending on its different availabilities across countries and local/national recommendations, could thus be a useful complementary investigation for assessing cognitive complaints.

Regarding the main objective of this study, brain ^18^F-FDG PET scans showed a good NPV for the risk of converting to dementia (85% for all neurodegenerative pathologies either with the NHDS or the NAB databases). This excellent NPV for the risk of conversion to dementia was also found when limited to MCI patients (84%). Moreover, neurodegenerative pathologies, in particular AD, can be ruled out with a negative brain PET scan due to the excellent NPV (81% for all neurodegenerative pathologies, 91% for AD); as observed in the analysis for dementia conversion, a similar conclusion could be reached when we restricted our analyses to MCI patients (NPVs of 78% and 88% for ruling out neurodegenerative pathologies and AD, respectively). In addition, survival analysis revealed that a brain ^18^F-FDG PET scan showing a neurodegenerative pattern was associated with a shorter time to dementia or death than was a normal brain PET scan. It should also be noted that brain ^18^F-FDG PET scans performed well in positively diagnosing neurodegenerative disorders, especially AD, with accuracies of 87%. Therefore, brain ^18^F-FDG PET is an interesting tool for the early diagnosis of neurodegenerative diseases to rule out a conversion to dementia or the diagnosis of a neurodegenerative disorder. In addition, brain ^18^F-FDG PET can be used to confirm a suspected diagnosis of AD.

Regarding the associated factors, age was the only predictive factor of dementia conversion identified for both the whole population and in a subanalysis restricted to patients whose PET scan suggested a neurodegenerative pattern. This latter finding is in agreement with the widespread recognition of age as a major factor influencing the risk of dementia conversion [22]. It is also important to note that a brain ^18^F-FDG PET scan favoring a diagnosis of AD also emerged as a risk factor predicting dementia conversion among the evaluated factors from the NHDS, but statistical analysis revealed that this relationship was not significant. Brain ^18^F-FDG PET scan was nevertheless the sole predictive factor of dementia conversion in the NAB database. By contrast, age under 60 years and/or a high level of education were protective factors, associated to brain ^18^F-FDG PET scan without any neurodegenerative pattern, suggesting that such patients would benefit less from this imaging modality. Interestingly, significant associations between the neurodegeneration pattern shown on brain ^18^F-FDG PET scans and the diagnosed stage of cognitive impairment and syndromic and etiological diagnoses were observed in this study. As expected, higher proportions of brain ^18^F-FDG PET scans without a neurodegenerative pattern were observed among SCI and MCI patients and among patients with an etiological diagnosis of subjective memory complaints, other causes, or psychiatric pathology. Interestingly, higher proportions of brain ^18^F-FDG PET scans without a neurodegenerative pattern were also observed in patients with syndromic dysexecutive or amnesic diagnoses, which can be explained by the predominant frontal involvement in dysexecutive disorders; this involvement is not easy to detect by brain ^18^F-FDG PET due to the high glycolytic uptake in the frontal lobes or to the limited involvement of the internal temporal areas in isolated amnesic syndromes [9].

In prospective studies including only MCI-AD-related patients [24, 25], high NPVs (94% and 95%, respectively) for identifying the risk of dementia conversion were reported similarly to our presented results. Other important studies investigating the diagnostic performance of brain ^18^F-FDG PET have employed postmortem autopsy analyses, the gold standard for identifying neurodegenerative disorders [26, 27]. Our results are also in line with those of previous studies, particularly in terms of the NPVs (near 80% for all our analyses versus 78% [26] and 84% [27]). One strength of our study, which was conducted with real-world data, is the large cohort of patients (more than 400 patients who were matched to at least one database) who were followed for at least three years. This large sample and the sufficient duration of follow-up are in accordance with the latest recommendations for promoting brain ^18^F-FDG PET as a biomarker for AD [15]. Moreover, our study has evaluated brain ^18^F-FDG PET with a combined visual and semi-quantitative analysis which is currently recommended in clinical practice [14]. Studies combining visual and semi-quantitative analyses to determine the predictive value of brain ^18^F-FDG PET for conversion to dementia are scarce in literature, and those of interest [28–30] were performed in relative low number of patients with the diagnostic follow-up only as a final outcome. Our study not only uses a large cohort of patients with real world data but has also investigated the predictive value of brain ^18^F-FDG PET with a combined visual and semi-quantitative analysis and with results of CSF biomarkers as an objective outcome for a part of the population matched with medical records. Based on these strengths, our current study thus confirms the potential role of brain ^18^F-FDG PET in the investigation of neurodegenerative disease.

Our study questions the current strategic place of brain ^18^F-FDG PET in the assessment of cognitive disorders in clinical practice. Brain ^18^F-FDG PET has been proposed within first-line investigations for the positive diagnosis of neurodegenerative disorders [10] or in association with amyloid PET depending on the risk of AD pathology [15]. One study confirmed that adding brain ^18^F-FDG PET to amyloid PET better predicted the long-term risk of conversion to dementia in amyloid-positive patients [31]. Obviously, the prescription of a brain ^18^F-FDG PET is highly dependent of national recommendations which can differ related to the availability of PET scanners. However, given our findings, we confirmed the potential place of brain ^18^F-FDG PET to rule out the risk of conversion to dementia in patients with cognitive disorders if the scan demonstrates a normal pattern. Excluding this risk of conversion early with high certainty allows better customization of patient management regimens, thus limiting global costs and ultimately minimizing economic burdens. A normal brain ^18^F-FDG PET scan is not rare in clinical practice; in the tertiary memory clinic of our department, approximately one-third (30%) of all patients treated by neurologist/geriatrician specialists present with normal brain PET findings. Of course, from a diagnostic or therapeutic perspective, the use of CSF biomarkers or amyloid or tau PET radiotracers will still be important keeping also in mind the recent development of plasma biomarkers which could dramatically change the lines of investigations regarding the diagnosis of neurodegenerative diseases [11, 32]. However, early PET imaging of the brain could serve as an adjunct examination to these techniques and may even be suitable on its own in areas where the resources highlighted here may not be available.

Nonetheless, our study has several limitations related to its retrospective and single-center nature. The criterion utilized in the NHDS to identify dementia conversion, uniquely based on the determination of an LTC for dementia, could underestimate the true number of patients who have converted to dementia but was chosen because of its good specificity. Moreover, the competitive risk of death on dementia conversion, despite the low number of patients who died, was evaluated for the secondary objectives but could not be evaluated for the main objective. Another limitation of the present study concerns the lack of confirmation of neurodegenerative diagnoses via postmortem autopsies. However, analyses of well-validated CSF biomarkers were performed as confirmation. Notably, there was also a possibility of slow-progressor patients, who could have biased our reported diagnostic performances because not all patients were followed up 5 years after they underwent the PET scan.

## Conclusion

The results obtained from a large cohort of consecutive patients with a history of memory complaints who underwent brain ^18^F-FDG PET prescribed by a specialist in a tertiary memory department within a clinical practice confirmed that brain ^18^F-FDG PET scans can rule out the risk of dementia conversion early with high certainty, allowing better customization of patient management regimens in the short term. These results, obtained through matching data to three real-world databases, could aid in positioning brain ^18^F-FDG PET as a useful tool for the assessment of neurodegenerative disorders taking into account cost-effectiveness of other concurrent techniques and local and national PET scanners availabilities. Additional multicentric studies pairing PET imaging data with patient data collected at a longer interval from the scan (e.g., 5 or 10 years) to further verify the prognostic value of brain PET studies in predicting the conversion to dementia could be implemented.

## Electronic supplementary material

Below is the link to the electronic supplementary material.

Additional file 1: Supplemental table 1: Data extracted from the National Health Data System (*n* = 403)

Additional file 2: Supplemental table 2: Bivariate analyses of factors predicting the risk of dementia conversion within the three years after the PET scan according to the National Health Data System (*n* = 403)

Additional file 3: Supplemental table 3: Bivariate analyses of factors predicting the risk of dementia conversion within the three years after the PET scan according to the National Health Data System in patients with a brain ^18^F-FDG PET scan demonstrating a pattern in favor of a neurodegenerative disease (*N* = 281)

Additional file 4: Supplemental table 4: Bivariate analyses of factors predictive of a brain ^18^F-FDG PET scan demonstrating a pattern not in favor of a neurodegenerative disease according to the National Health Data System (*n* = 403)

## Data Availability

No datasets were generated or analysed during the current study.

## Abbreviations

18F-FDG: [18 F] fluoro-2-deoxy-2-D-glucose
PET: Positron Emission Tomography
NHDS: National Health Data System
NAB: National Alzheimer Bank
LP: Lumbar puncture
CSF: Cerebrospinal fluid
AD: Alzheimer’s disease
SCI: Subjective cognitive impairment
MCI: Mild cognitive impairment
MRI: Magnetic resonance imaging
CGMr: Glucose metabolic rate
CMRR: Centre Mémoire de Ressources et de Recherche
CHRU: Centre Hospitalier Régional Universitaire
CESREES: Comité Ethique et Scientifique pour les Recherches, les Etudes et les Evaluation en Santé
CNIL: Commission Nationale Informatique et Libertés
OSEM: Ordered Subset Expectation Maximization
PSF: Point Spread Function
SPM: Statistical Parametric Mapping
FWHM: Full-Width at Half-Maximum
ADNI: Alzheimer’s Disease Neuroimaging Initiative
LTC: Long-term condition
Se: Sensitivity
Sp: Specificity
Acc: Accuracy
PPV: Positive Predictive Value
NPV: Negative Predictive Value
