# Correction: ^18^F-FDG PET can effectively rule out conversion to dementia and the presence of CSF biomarker of neurodegeneration: a real-world data analysis

**DOI:** 10.1186/s13195-024-01608-3

**Published:** 2024-11-13

**Authors:** Sébastien Heyer, Maïa Simon, Matthieu Doyen, Ali Mortada, Véronique Roch, Elodie Jeanbert, Nathalie Thilly, Catherine Malaplate, Anna Kearney-Schwartz, Thérèse Jonveaux, Aurélie Bannay, Antoine Verger

**Affiliations:** 1 Department of Nuclear Medicine and Nancyclotep Imaging Platform, Université de Lorraine, CHRU Nancy, Nancy, F-54000 France; 2 Department of Methodology, Promotion and Investigation, Université de Lorraine, CHRU-Nancy, Nancy, F-54000 France; 3 Université de Lorraine, IADI, INSERM U1254, Nancy, F-54000 France; 4 Department of Biochemistry, Université de Lorraine, CHRU-Nancy, Nancy, F-54000 France; 5 Department of Geriatrics, Université de Lorraine, CHRU-Nancy, Nancy, F-54000 France; 6 CMRR, University Hospital Nancy, Nancy, F-54000 France; 7 Department of Neurology, University Hospital Nancy, Nancy, F-54000 France; 8 Medical Assessment and Information Department, Université de Lorraine, CHRU-Nancy, Nancy, 54000 France

**Correction:** ***Alz Res Therapy*** **16, 182 (2024)**

10.1186/s13195-024-01535-3

Following publication of the original article [1], corrections were made to the three numbers (1), (2), (3) in Methods section of Abstract that were incorrectly presented as reference citations: (Prince M, Wimo A, Guerchet Maëlenn, Ali G-C, Wu Y-T et al. World Alzheimer Report 2015. The Global Impact of Dementia: An analysis of prevalence, incidence, cost and trends. [Research Report] Alzheimer’s Disease International. 2015. 2015.); (Jack CR, Bennett DA, Blennow K, Carrillo MC, Dunn B, Haeberlein SB, et al. NIA-AA Research Framework: Toward a biological definition of Alzheimer’s disease. Alzheimers Dement. 2018;14(4):535–62.) and (Davis M, O`Connell T, Johnson S, Cline S, Merikle E, Martenyi F, et al. Estimating Alzheimer’s Disease Progression Rates from Normal Cognition Through Mild Cognitive Impairment and Stages of Dementia. CAR. 2018;15(8):777–88.).

The original article [1] has been updated.
