# Supplementary material for: 18F-FDG PET can effectively rule out conversion to dementia and the presence of CSF biomarker of neurodegeneration: a real-world data analysis
Source: Alzheimers Res Ther. 2024 Aug 13;16:182. doi: 10.1186/s13195-024-01535-3 (PMC11320856; doi:10.1186/s13195-024-01535-3)
Supplement: Supplementary file 1 — Additional file 1: Supplemental table 1: Data extracted from the National Health Data System (n = 403) [file 13195_2024_1535_MOESM1_ESM.docx]

**Supplemental Table 1.** Data extracted from the National Health Data System (n=403).

| Variable | Conditions | n (%) |
| --- | --- | --- |
| Onset of dementia within the three years after the PET scan (LTC for dementia within the three years after the PET scan) | No | 298 (73.95%) |
|  | Yes | 105 (26.05%) |
| At least one hospitalization from any cause within the three years after the PET scan | No | 49 (12.16%) |
|  | Yes | 354 (87.84%) |
| Death within the three years following the PET scan | No | 339 (84.12%) |
|  | Yes | 64 (15.88%) |
| History of LTC: high blood pressure* | No | 403 (100.00%) |
|  | Yes | 0 (0.00%) |
| History of LTC: diabetes* | No | 385 (98.01%) |
|  | Yes | 8 (1.99%) |
| History of LTC: stroke* | No | 395 (98.01%) |
|  | Yes | 8 (1.99%) |
| History of LTC: Parkinson's disease* | No | 375 (93.05%) |
|  | Yes | 28 (6.95%) |
| History of LTC: psychiatric conditions* | No | 382 (94.79%) |
|  | Yes | 21 (5.21%) |
| History of LTC: vasculitis* | No | 401 (99.50%) |
|  | Yes | 2 (0.50%) |
| Previous neuropsychiatric hospitalization* | No | 135 (33.50%) |
|  | Yes | 268 (66.50%) |
| Previous anxiolytic treatment* | No | 220 (54.59%) |
|  | Yes | 183 (45.41%) |
| Previous antidepressant treatment* | No | 208 (51.61%) |
|  | Yes | 195 (48.39%) |
| Previous anti-dementia treatment* | No | 397 (98.51%) |
|  | Yes | 6 (1.49%) |
| Previous hypnotic treatment* | No | 295 (73.20%) |
|  | Yes | 108 (26.80%) |
| Previous anti-psychotic treatment* | No | 356 (88.34%) |
|  | Yes | 47 (11.66%) |

LTC: long-term condition, PET: positron emission tomography; * *At least one in the three years preceding the PET scan*
