# Supplementary material for: 18F-FDG PET can effectively rule out conversion to dementia and the presence of CSF biomarker of neurodegeneration: a real-world data analysis
Source: Alzheimers Res Ther. 2024 Aug 13;16:182. doi: 10.1186/s13195-024-01535-3 (PMC11320856; doi:10.1186/s13195-024-01535-3)
Supplement: Supplementary file 2 — Additional file 2: Supplemental table 2: Bivariate analyses of factors predicting the risk of dementia conversion within the three years after the PET scan according to the National Health Data System (n = 403) [file 13195_2024_1535_MOESM2_ESM.docx]

**Supplemental Table 2.** Bivariate analyses of factors predicting the risk of dementia conversion within the three years after the PET scan according to the National Health Data System (n=403).

| Variable | Conditions | Patients with dementia within 3 years (N=105)  n (%) | Hazard ratio | Confidence interval | p value |
| --- | --- | --- | --- | --- | --- |
|  |  |  |  |  |  |
| Age |  |  | 1.046 | [1.028; 1.065] | **< 0.0001** |
| Sex | Men | 54 (51.43%) | ref | ref | - |
|  | Women | 51 (48.57%) | 1.258 | [0.859; 1.843] | 0.238 |
| Level of education | Primary school | 26 (30.23%) | 2.439 | [1.110; 5.359] | **0.027** |
|  | College | 11 (12.79%) | 2.223 | [0.907; 5.446] | 0.081 |
|  | Youth training NVQ (National Vocational Qualification) | 16 (18.60%) | 1.665 | [0.715; 3.877] | 0.237 |
|  | High school | 8 (9.30%) | ref | ref | - |
|  | Graduate studies | 25 (29.07%) | 1.786 | [0.815; 3.915] | 0.147 |
|  | Missing data | 19 | - | - | - |
| PET diagnosis of neurodegenerative disease | No | 18 (17.14%) | ref | ref | - |
|  | Yes | 87 (82.86%) | 2.416 | [1.475; 3.957] | **0.001** |
| PET diagnosis of Alzheimer's disease | No | 61 (58.10%) | ref | ref | - |
|  | Yes | 44 (41.90%) | 2.384 | [1.615; 3.520] | **< 0.0001** |
| Normal PET | No | 87 (82.86%) | ref | ref | - |
|  | Yes | 18 (17.14%) | 0.425 | [0.259; 0.695] | **0.001** |
| History of LTC: diabetes | No | 98 (93.33%) | ref | ref | - |
|  | Yes | 7 (6.67%) | 1.609 | [0.770; 3.363] | 0.206 |
| Previous neuropsychiatric hospitalization | No | 36 (34.29%) | ref | ref | - |
|  | Yes | 69 (65.71%) | 0.980 | [0.658; 1.461] | 0.922 |
| Previous anxiolytic treatment | No | 61 (58.10%) | ref | ref | - |
|  | Yes | 44 (41.90%) | 0.861 | [0.585; 1.269] | 0.451 |
| Previous antidepressant treatment | No | 52 (49.52%) | ref | ref | - |
|  | Yes | 53 (50.48%) | 1.111 | [0.758; 1.626] | 0.590 |
| Previous hypnotic treatment | No | 78 (74.29%) | ref | ref | - |
|  | Yes | 27 (25.71%) | 0.954 | [0.615; 1.481] | 0.834 |
| Previous anti-psychotic treatment | No | 99 (94.29%) | ref | ref | - |
|  | Yes | 6 (5.71%) | 0.417 | [0.184; 0.944] | **0.036** |

*LTC: long-term condition, PET: positron emission tomography*
