# Supplementary material for: 18F-FDG PET can effectively rule out conversion to dementia and the presence of CSF biomarker of neurodegeneration: a real-world data analysis
Source: Alzheimers Res Ther. 2024 Aug 13;16:182. doi: 10.1186/s13195-024-01535-3 (PMC11320856; doi:10.1186/s13195-024-01535-3)
Supplement: Supplementary file 3 — Additional file 3: Supplemental table 3: Bivariate analyses of factors predicting the risk of dementia conversion within the three years after the PET scan according to the National Health Data System in patients with a brain 18F-FDG PET scan demonstrating a pattern in favor of a neurodegenerative disease (N = 281) [file 13195_2024_1535_MOESM3_ESM.docx]

**Supplemental Table 3.** Bivariate analyses of factors predicting the risk of dementia conversion within the three years after the PET scan according to the National Health Data System in patients with a brain ^18^F-FDG PET scan demonstrating a pattern in favor of a neurodegenerative disease (N=281).

| Variable | Conditions | Patients with dementia within 3 years (N=87)  n (%) | | Hazard ratio | Confidence interval | p value |
| --- | --- | --- | --- | --- | --- | --- |
| Age |  |  | | 1.032 | [1.010; 1.054] | **0.005** |
| Sex | Men | 46 (52.87%) | | ref | ref | - |
|  | Women | 41 (47.13%) | | 1.099 | [0.720; 1.672] | 0.660 |
| Level of education | Primary school | | 22 (31.43%) | 1.649 | [0.678; 4.012] | 0.270 |
|  | College | | 8 (11.43%) | 1.501 | [0.530; 4.255] | 0.445 |
|  | Youth training NVQ (National Vocational Qualification) | | 15 (21.43%) | 1.453 | [0.570; 3.705] | 0.434 |
|  | High school | | 6 (8.57%) | ref | ref | - |
|  | Graduate studies | | 19 (27.14%) | 1.545 | [0.629; 3.792] | 0.342 |
|  | Missing data | | 17 | - | - | - |
| History of LTC for diabetes | No | 81 (93.10%) | | ref | ref | - |
|  | Yes | 6 (6.90%) | | 1.554 | [0.730; 3.307] | 0.253 |
| Previous neuropsychiatric hospitalization | No | 31 (35.63%) | | ref | ref | - |
|  | Yes | 56 (64.37%) | | 0.812 | [0.527; 1.253] | 0.347 |
| Previous anxiolytic treatment | No | 48 (55.17%) | | ref | ref | - |
|  | Yes | 39 (44.83%) | | 1.040 | [0.683; 1.584] | 0.855 |
| Previous antidepressant treatment | No | 41 (47.13%) | | ref | ref | - |
|  | Yes | 46 (52.87%) | | 1.197 | [0.787; 1.821] | 0.400 |
| Previous hypnotic treatment | No | 66 (75.86%) | | ref | ref | - |
|  | Yes | 21 (24.14%) | | 1.010 | [0.617; 1.653] | 0.968 |
| Previous anti-psychotic  treatment | No | 84 (96.55%) | | ref | ref | - |
|  | Yes | 3 (3.45%) | | 0.239 | [0.076; 0.750] | **0.014** |

*LTC: long-term condition, PET: positron emission tomography*
