# Supplementary material for: 18F-FDG PET can effectively rule out conversion to dementia and the presence of CSF biomarker of neurodegeneration: a real-world data analysis
Source: Alzheimers Res Ther. 2024 Aug 13;16:182. doi: 10.1186/s13195-024-01535-3 (PMC11320856; doi:10.1186/s13195-024-01535-3)
Supplement: Supplementary file 4 — Additional file 4: Supplemental table 4: Bivariate analyses of factors predictive of a brain 18F-FDG PET scan demonstrating a pattern not in favor of a neurodegenerative disease according to the National Health Data System (n = 403) [file 13195_2024_1535_MOESM4_ESM.docx]

**Supplemental Table 4.** Bivariate analyses of factors predictive of a brain ^18^F-FDG PET scan demonstrating a pattern not in favor of a neurodegenerative disease according to the National Health Data System (n=403).

| Variable | Conditions | Patients with normal PET scans (N=120)  n (%) | Odds ratio | Confidence interval | p value |
| --- | --- | --- | --- | --- | --- |
| Age | < 60 years | 48 (40.00%) | 7.345 | [3.604; 14.966] | **< 0.0001** |
|  | 60–69 years | 30 (25.00%) | 1.752 | [0.881; 3.484] | 0.110 |
|  | 70-79 years | 26 (21.67%) | 1.078 | [0.540; 2.152] | 0.831 |
|  | > 80 years | 16 (13.33%) | ref | ref | **-** |
| Sex | Men | 71 (59.17%) | ref | ref | **-** |
|  | Women | 49 (40.83%) | 0.836 | [0.542; 1.288] | 0.416 |
| Level of education | Primary school | 15 (14.42%) | ref | ref | **-** |
|  | College | 10 (9.62%) | 1.722 | [0.680; 4.359] | 0.251 |
|  | Youth training NVQ (National Vocational Qualification) | 16 (15.38%) | 1.350 | [0.608; 2.997] | 0.4614 |
|  | High school | 25 (24.04%) | 4.133 | [1.874; 9.115] | **0.0004** |
|  | Graduate studies | 38 (36.54%) | 2.586 | [1.419; 5.747] | **0.003** |
|  | Missing data | 16 | - | - | - |
| History of LTC for diabetes | No | 115 (95.83%) | ref | ref | **-** |
|  | Yes | 5 (4.17%) | 0.903 | [0.315; 2.591] | 0.850 |
| History of LTC for Parkinson's disease | No | 112 (93.33%) | ref | ref | **-** |
|  | Yes | 8 (6.67%) | 0.939 | [0.402; 2.196] | 0.885 |
| History of LTC for psychiatric conditions | No | 115 (95.83%) | ref | ref | **-** |
|  | Yes | 5 (4.17%) | 0.726 | [0.260; 2.028] | 0.541 |
| Previous neuropsychiatric hospitalization | No | 46 (38.33%) | ref | ref | **-** |
|  | Yes | 74 (61.67%) | 0.738 | [0.473; 1.152] | 0.181 |
| Previous anxiolytic treatment | No | 62 (51.67%) | ref | ref | **-** |
|  | Yes | 58 (48.33%) | 1.182 | [0.771; 1.814] | 0.443 |
| Previous antidepressant treatment | No | 63 (52.50%) | ref | ref | **-** |
|  | Yes | 57 (47.50%) | 0.951 | [0.620; 1.458] | 0.817 |
| Previous hypnotic treatment | No | 81 (67.50%) | ref | ref | **-** |
|  | Yes | 39 (32.50%) | 1.493 | [0.935; 2.386] | 0.094 |
| Previous anti-psychotic treatment | No | 105 (87.50%) | ref | ref | **-** |
|  | Yes | 15 (12.50%) | 1.121 | [0.582; 2.156] | 0.733 |

*LTC: long-term condition, PET: positron emission tomography*
